# Reducing Cross-Sensor Domain Gaps in Tactile Sensing via Few-Sample-Driven Style-to-Content Unsupervised Domain Adaptation

**DOI:** 10.3390/s25010256

**Published:** 2025-01-05

**Authors:** Xingshuo Jing, Kun Qian

**Affiliations:** 1 School of Automation, Southeast University, Nanjing 210096, China; jingsdjnyt@seu.edu.cn; 2 Key Laboratory of Measurement and Control of Complex Systems of Engineering, Ministry of Education, Nanjing 210096, China; 3 Southeast University Shenzhen Research Institute, Shenzhen 518063, China

**Keywords:** cross-sensor domain gaps, tactile sensing, unsupervised domain adaptation, style to content

## Abstract

Transferring knowledge learned from standard GelSight sensors to other visuotactile sensors is appealing for reducing data collection and annotation. However, such cross-sensor transfer is challenging due to the differences between sensors in internal light sources, imaging effects, and elastomer properties. By understanding the data collected from each type of visuotactile sensors as domains, we propose a few-sample-driven style-to-content unsupervised domain adaptation method to reduce cross-sensor domain gaps. We first propose a Global and Local Aggregation Bottleneck (GLAB) layer to compress features extracted by an encoder, enabling the extraction of features containing key information and facilitating unlabeled few-sample-driven learning. We introduce a Fourier-style transformation (FST) module and a prototype-constrained learning loss to promote global conditional domain-adversarial adaptation, bridging style-level gaps. We also propose a high-confidence guided teacher–student network, utilizing a self-distillation mechanism to further reduce content-level gaps between the two domains. Experiments on three cross-sensor domain adaptation and real-world robotic cross-sensor shape recognition tasks demonstrate that our method outperforms state-of-the-art approaches, particularly achieving 89.8% accuracy on the DIGIT recognition dataset.

## 1. Introduction

**Figure 1:**
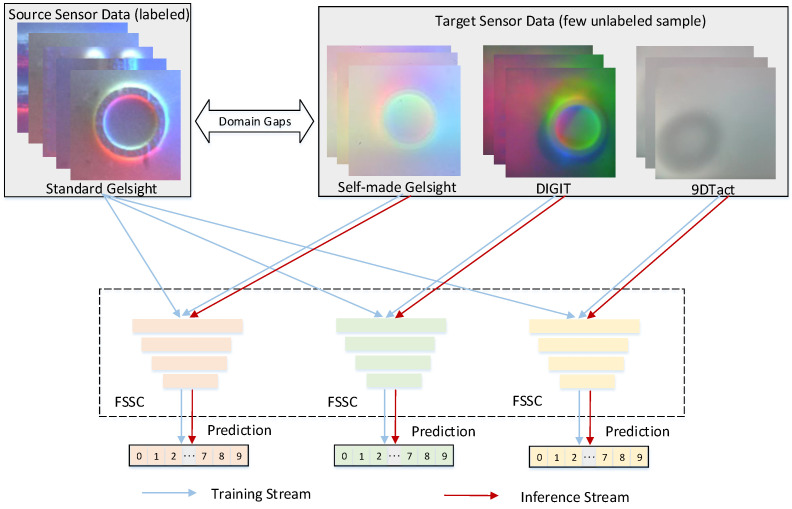
Overview of the cross-sensor domain adaptation pipeline. Sufficient labeled data from the standard GelSight tactile sensor and a few unlabeled target domain samples, either from a self-made GelSight sensor, a DIGIT sensor, or a 9DTact tactile sensor, are used to train our proposed FSSC. The trained model is then deployed on the tactile sensor corresponding to the target domain.

Traditional robotic tactile perception methods [1,2,3] typically rely on the collection of large-scale real-world labeled data for training, which is both resource-intensive and accelerates wear on tactile sensors. With the advancement of tactile sensors like GelSight [4] and the availability of extensive labeled data, an appealing alternative is to utilize the sufficient available data from the standard GelSight sensor to train models and generalize them to other similar types of tactile sensors, as illustrated in Figure 1. However, due to significant visuotactile domain gaps, direct deployment across sensors often results in substantial performance reduction.

One promising solution to overcome the domain gaps is domain adaptation [5,6,7,8]. However, compared to existing domain adaptation, cross-sensor domain adaptation transfer faces greater challenges due to larger domain gaps. As shown in Figure 1, there are significant gaps between the tactile images captured by the standard GelSight sensor and those captured by other tactile sensors. First, gaps in the internal lighting sources of the sensors result in noticeable variations in the external styles of the images, with these gaps primarily existing at the low-frequency level. Second, even after excluding the aforementioned style gaps, inconsistencies in contextual content can still arise due to the elastomer properties of the sensors, such as variations in foreground shadows and edge smoothness. Therefore, addressing this larger domain gap and achieving cross-sensor domain adaptation poses a key challenge.

Moreover, acquiring tactile images can be even more costly than the labeling process itself in some recognition tasks. Although most domain adaptation methods [5,6,7,8] are independent of sample labeling, they still require a large number of target domain samples for adaptation training, which also accelerates sensor wear. This trade-off between data acquisition and sensor longevity highlights the limitations of existing approaches. Consequently, achieving effective recognition knowledge transfer between different sensors using a limited amount of target domain data remains another meaningful challenge.

In this paper, we propose a few-sample-driven style-to-content unsupervised domain adaptation method, named FSSC, to reduce the domain gap between different real-world tactile sensors. By hierarchically adapting the style and content information of the source and target domains, this method can effectively leverage a few unlabeled target domain samples to reduce the data distribution gap between two sensors and ensure robust feature extraction for the target domain samples. We construct an FST module and introduce a prototype network to extract class constraints, enabling style adaptation through global conditional domain-adversarial learning. In addition, the self-supervised teacher–student knowledge distillation mechanism refines the learning process, allowing the student model to further align global features across both domains, aiming to achieve content-level knowledge transfer.

Our contributions include the following:

- We propose a few-sample-driven style-to-content cross-sensor domain adaptation framework (FSSC) for the first time in tactile recognition tasks, which joins global conditional adversarial style-level learning and self-distillation-based content-level learning to bridge cross-sensor domain gaps.
- A GLAB layer is proposed to compress encoded features, filtering for features containing key information and facilitating few-sample-driven learning.
- We introduce an FST module and design a prototype-constrained loss, allowing for feature alignment in the few-sample target domain while ensuring robust classification in the source domain.
- Extensive experiments on three constructed cross-sensor domain adaptation and robotic tactile shape recognition tasks validate that the proposed algorithm outperforms state-of-the-art methods, highlighting its applicability in robotic tactile recognition tasks.

The remainder of this paper is organized as follows: Section 2 provides an overview of the related literature. In Section 3, we present our proposed method, detailing the overall architecture, key modules, and the optimization objective. Section 4 contains detailed experiments and analysis. Finally, Section 5 provides further discussion and conclusions for this paper.

## 2. Related Work

### 2.1. Optical Tactile Sensors

Optical tactile sensors [9,10,11,12,13,14,15] have been widely used in the field of robotic perception due to their high resolution and precise surface contact measurement capabilities. These sensors capture tactile information using optical systems, making them particularly suitable for tasks requiring detailed surface analysis, such as texture recognition, shape recognition, and object manipulation. Currently, these sensors can be categorized into marker-based tactile sensors [9,10,11,12] and image-based tactile sensors [13,14,15,16,17]. This paper focuses on image-based sensors as they are suited for capturing fine textures.

The representative GelSight sensor, first proposed by Johnson et al. [4], provides high-resolution tactile information by capturing images of the deformation of an elastomeric coating upon contact. GelSight’s outstanding performance in capturing surface details has made it widely used in tasks such as grasp stability prediction and object recognition, making it one of the most well-established tactile sensors to date. The DIGIT tactile sensor [18], which shares similar optical principles with GelSight, is more compact and is also extensively employed for perceiving object shape, texture, and contact force information. Recently, the 9DTact tactile sensor [19] has extended traditional tactile sensing by offering nine-dimensional tactile information, including 3D shape reconstruction and generalizable 6D force estimation. It integrates optical sensing and a soft contact surface, enabling the capture of high-fidelity tactile information during complex surface interactions.

These optical sensors are prone to damage, and repeated experimental operations can easily degrade or break the sensor membrane. Consequently, many studies have attempted to train perception models using simulators and then deploy them in real-world environments to avoid large-scale real-world data collection and annotation. However, some tactile sensors may lack the corresponding simulator support, which poses significant limitations for sim-to-real transfer. This paper explores the similarities in imaging among tactile sensors, using the standard GelSight sensor as a benchmark to transfer recognition models across different real-world tactile sensors, thereby reducing the wear and tear on tactile sensors.

### 2.2. Reducing Cross-Sensor Domain Gaps for Tactile Sensing

Many studies have focused on reducing the significant performance drop that occurs when deploying perception models trained in simulators directly to real-world scenarios by reducing the domain gap in tactile perception. Currently, common methods for minimizing domain gaps can be categorized into domain randomization-based methods [4,20,21] and domain adaptation-based methods [7,22,23,24,25,26,27,28,29,30,31,32,33,34,35,36,37,38].

Domain randomization-based methods [4,20,21] either randomize object colors, lighting, and physical parameters in the simulator or apply randomized noise processing, lighting variations, and geometric transformations to the captured simulated data. However, the data distribution generated through domain randomization can be challenging to align with real-world environments. In other words, a significant portion of the randomized data may be ineffective or redundant. On the other hand, domain adaptation-based methods [7,22,23,24,25,26,27,28,29,30,31,32,33,34,35,36] offer greater flexibility by learning mappings between the two data distributions at either the feature level or the pixel level, thereby achieving fine-grained adaptation between two domains. For instance, Chen et al. [7] propose using CycleGAN to reduce the domain gap between simulated and real-world data for the GelSight tactile sensor. Jing et al. [5] further introduce an adaptive correlation attention mechanism and a task-related constraint to enhance tactile image generation performance. Tang et al. [39] propose a novel method for single-sample-driven and multi-source sim-to-real tactile information understanding. Recently, some self-supervised approaches [40,41,42] have also employed pseudo-labels to iteratively optimize the network, achieving outstanding results even in label-free scenarios.

However, these domain adaptation methods are primarily effective for addressing cases with relatively small domain gaps or similar styles, such as domain adaptation between simulation and real-world data. When the domain gap is larger, such as between different tactile sensors, there are significant differences in imaging color styles and content patterns, including the perception of touch surface details, which pose considerable limitations. In contrast, we decouple style and content in domain gaps and design a domain-adversarial and self-supervised progressive training framework, which significantly alleviates the domain gap between different sensors.

## 3. Methodology

In this section, we first introduce the overall framework of the proposed FSSC method. Then, we present the key modules involved in the two pipelines. Finally, the overall optimization loss is described.

### 3.1. Overview

**Figure 2:**
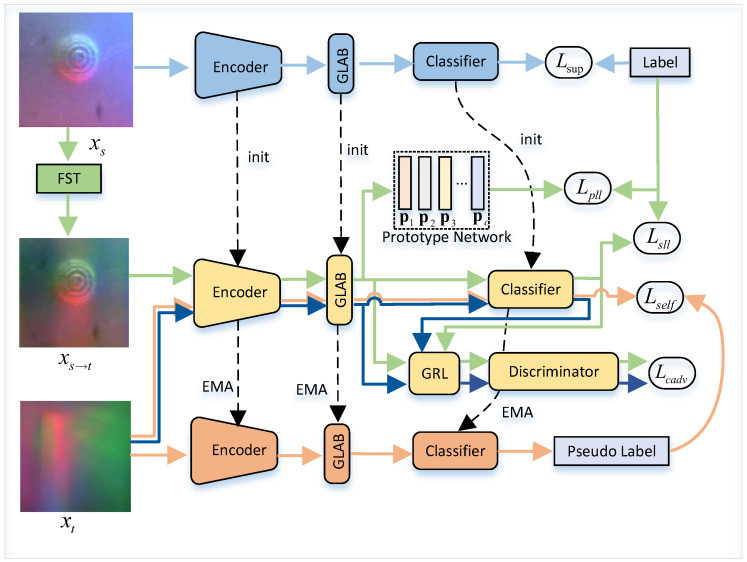
The overall framework of our proposed FSSC.

As shown in Figure 2, the training of the proposed FSSC method is divided into three parts: pre-training, style adaptation, and content adaptation. For each part, our shape recognition network consists of an encoder, a bottleneck layer, and a classifier. During the training phase, labeled source domain data are first used for pre-training the model. Then, the FST module is used to transform the source domain data into the target domain style, and domain adversarial training based on a prototypical network is performed using both the transformed data and a small amount of unlabeled target domain data. Finally, content adaptation is further enabled through high-confidence guided teacher–student knowledge distillation.

### 3.2. Tactile Shape Recognition Network

We construct a tactile shape recognition network and apply it to the pre-training, style adaptation, and content adaptation phases. This network comprises three main components: an encoder, a bottleneck layer, and a classifier, with tactile images $x\in R^{H\times W\times 3}$ as input. We adopt the feature extraction layers of the Swin Transformer (SwinT) [43] as the encoder, primarily due to SwinT’s strong global context extraction capability along with its ability to capture local features. This allows for the acquisition of richer semantic information.

**Figure 3:**
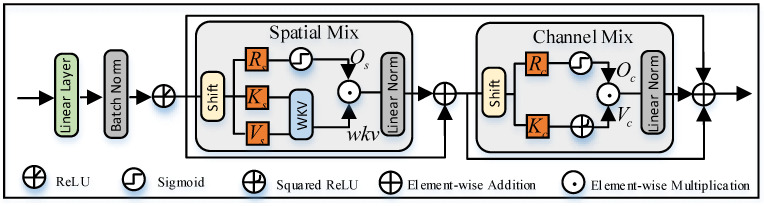
Global and Local Aggregation Bottleneck, i.e., GLAB.

The design of the bottleneck layer focuses on feature compression and information aggregation. The bottleneck layer compresses high-dimensional features and aggregates global context with local details, enabling the model to effectively reduce redundant information. Global information aids in recognizing macro shape features, while the local aggregation layer extracts details such as edges and corners, thereby improving classification accuracy. Specifically, as shown in Figure 3, we first apply a linear layer to compress the features obtained from the encoder, followed by batch normalization and ReLU activation. Then, based on the original RWKV [44] architecture, we integrate a spatial mixing module and a channel mixing module to efficiently extract and fuse temporal and spatial information. The spatial mixing module functions as an attention mechanism, performing global attention computation with linear complexity, while the channel mixing module, acting as a Feed-Forward Network (FFN), conducts feature fusion along the channel dimension. Notably, to extend the RWKV architecture, which is tailored for processing 1D sequences, to 2D tactile images, we first increase the upper limit of WKV [45] attention from *t* (the current token) to T−1 (the last token), ensuring that all tokens are bidirectionally visible during computation to better capture global dependencies. Additionally, we utilize a multi-branch convolutional weighting approach to shift and merge adjacent tokens from various directions, with each branch responsible for shifting tokens within a specific contextual range. Given an input feature $z\in R^{H\times W\times C}$, the fusion process can be represented as follows:

(1) $$\begin{array}{c} \mathrm{Shift}\left(z\right)=\eta_{1}\mathrm{DCon}v_{5\times 5}\left(z\right)+\eta_{2}\mathrm{DCon}v_{3\times 3}\left(z\right)+\eta_{3}\mathrm{DCon}v_{1\times 1}\left(z\right)+\eta_{4}z \end{array}$$

where $\eta_{i}$ represents a learnable fusion factor, and ${\mathrm{DConv}}_{k\times k}$ denotes a depthwise convolution with a kernel size of k×k. Specifically, during the testing phase, we use only the 5×5 convolution branch for shifting. This design enables the model to fully leverage the inherent spatial relationships in 2D tactile images, capturing effective local contextual information.

For the classifier, we employ a single fully connected layer for classification, as the bottleneck features already exhibit strong linear classification capability, eliminating the need for more complex classification structures. Additionally, the supervised pre-training loss $L_{pre}$ on the source domain data for this structure can be represented as follows:

(2) $$\begin{array}{c} L_{pre}=-\frac{1}{N_{s}}\sum_{i=1}^{N_{s}}{y_{s,i}}^{T}\log G_{c}\left(x_{s,i}\right) \end{array}$$

where $N_{s}$ denotes the total number of source domain samples, $x_{s,i}$ and $y_{s,i}$ represent the *i*-th sample and its ground truth label, respectively, and $G_{c}\left(\cdot \right)$ is the classifier output. This loss is obtained by averaging the cross-entropy loss across all source domain samples.

### 3.3. Prototype-Based Cross-Style Domain Adversarial Learning

To enable effective stylized learning, we first introduce the Fourier transform to reduce the style distance between the source and target domains. Then, we use a prototypical network to constrain the model’s learning between classes, while constructing a conditional domain-adversarial mechanism to extract domain-invariant features, enhancing cross-domain classification capability at the style level.

#### 3.3.1. Fourier-Style Transformation

**Figure 4:**
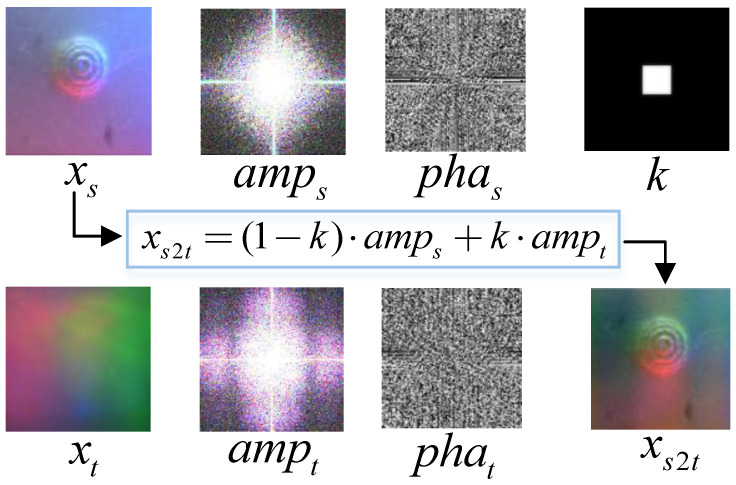
The process of Fourier-style transformation. $x_{s}$ and $x_{t}$ represent the real-world data from the source and target domains, respectively. amp and pha denote the amplitude and phase calculated from the corresponding real-world data. *k* represents the modified mask used for the Fourier transform.

We use the Fourier transform to process source domain images in the frequency domain, thereby reducing the style distance with the target domain. We consider the primary style-influencing factor to be the low-frequency background texture occupying most of the image, which is mainly affected by differences in internal lighting within the sensors. As shown in Figure 4, we extract the amplitude and phase maps of both the source and target domains, then replace the source domain amplitude map with that of the target domain using a rectangular mask, whose value is zero except for the center region where lb∈(0,1). The mask *k* can be represented as follows:

(3) $$\begin{array}{c} k\left(h,w\right)=\left\{\begin{array}{cc} 1, & \mathrm{if}\;-lb\cdot H\leq h\leq lb\cdot H\;\mathrm{and}\;-lb\cdot W\leq w\leq lb\cdot W,\\ 0, & \mathrm{otherwise}. \end{array}\right. \end{array}$$

where we assume the center of the image is at (0,0). Note that this is not measured in pixels, so the choice of the kernel scale factor lb is independent of the image size or resolution.

This operation aligns the source and target domains at the low-frequency level, enhancing the model’s ability to extract stylized features. In particular, we apply smoothing to the edges of the central rectangular mask to achieve a smooth style transition between the source and target amplitudes, avoiding inconsistent dark spots caused by abrupt amplitude changes around the mask edges. Additionally, we use the Fourier transform for frequency domain processing and then apply an inverse Fourier transform to restore the image to match the original input.

#### 3.3.2. Prototypical Constraint Learning

In our experiments, we observed that features from different classes can overlap under some conditions, which is detrimental to multi-class tasks. Therefore, we define a series of prototype vectors $\left\{p_{1},p_{2},\ldots ,p_{n_{c}}\right\}$, where $n_{c}$ denotes the number of classes, and each prototype corresponds to a specific class. Specifically, for each batch of samples, we calculate the mean feature of each class and update it using an Exponential Moving Average (EMA) with a smoothing coefficient α as training progresses. This update strategy requires no additional training parameters and ensures model stability. Our primary goal with this design is to guide the model toward learning more discriminative feature representations by defining a set of prototypical constraints. During training, distance constraints are introduced to ensure that samples are closer to their respective class prototype and further from prototypes of other classes.

The prototype vectors serve as representative features for their respective classes, and we use the l−2 norm to compute the distance between analogous-target domain features and all class prototypes. The distance between a feature and a prototype vector is calculated as follows:

(4) $$\begin{array}{c} \tau \left(x_{s2t,i},p_{m}\right)={∥x_{s2t,i}-p_{m}∥}_{2} \end{array}$$

where $x_{s2t,i}$ denotes the *i*-th style-transformed source domain sample, i.e., the analogous-target domain sample, and $p_{m}$ represents the prototype vector of the *m*-th class. To facilitate loss optimization, we apply softmax to the negative distances between the sample and each class prototype, resulting in $\tilde{\tau }\left(x_{s2t,i},p_{m}\right)$. Consequently, we obtain the distance vector:

(5) $$\begin{array}{c} d_{s2t,i}={\left[\tilde{\tau }\left(x_{s2t,i},p_{1}\right),\tilde{\tau }\left(x_{s2t,i},p_{2}\right),\ldots ,\tilde{\tau }\left(x_{s2t,i},p_{nc}\right)\right]}^{T} \end{array}$$

We then use cross-entropy to optimize the prototype constraint loss $L_{pll}$, represented as follows:

(6) $$\begin{array}{c} L_{pll}=-\frac{1}{N_{s2t}}\sum_{i=1}^{N_{s2t}}{y_{s2t,i}}^{T}\log d_{s2t,i} \end{array}$$

where $N_{s2t}$ and $y_{s2t,i}$ denote the total number of analogous-target domain samples and the *i*-th sample, respectively. This loss indicates that when the constraint loss is minimized, the distance between the *i*-th sample and the prototype vector of its true class is minimized, while the distance to other class prototypes is maximized. By optimizing under this constraint, the prototypical network not only reduces feature overlap between classes, thereby improving classification performance, but also enhances adaptability to unseen classes or sparse samples, especially in few-shot learning scenarios.

#### 3.3.3. Global-Level Conditional Feature Alignment

To alleviate style shifts, we perform global-level conditional feature alignment to align features between the two domains. As shown in Figure 2, inspired by the global memory mechanism in the Transformer encoder, we utilize the global information extracted by the encoder to construct unsupervised domain alignment. We extract the class token features of both domains, output by GLAB, as global features, i.e., $G_{b}\left(x_{s2t}\right)\in R^{768\times 1}$ and $G_{b}\left(x_{t}\right)\in R^{768\times 1}$. Using conditional domain-adversarial methods, we introduce a gradient reversal layer and class distribution learning, and treat the joint distribution of features and class predictions as the adversarial objective to extract global-level domain-invariant features. Thus, during the adversarial learning process, the global feature distributions of the two domains are aligned. Additionally, by leveraging class information, the conditional distributions for each class in the analogous-target and target domains also match, especially when the class distributions between the two domains are significantly different.

Specifically, we jointly represent the output of the bottleneck layer $G_{b}\left(x\right)$ and the class prediction $G_{c}\left(x\right)$, generating a joint representation by computing the outer product of the features and class predictions as follows:

(7) $$\begin{array}{cc} Z_{s2t,i} & =G_{b}\left(x_{s2t,i}\right)⊗G_{c}\left(x_{s2t,i}\right)\\ Z_{t,i} & =G_{b}\left(x_{t,i}\right)⊗G_{c}\left(x_{t,i}\right) \end{array}$$

Then, the conditional adversarial loss $L_{cadv}$ is given by the following:

(8) $$\begin{array}{c} L_{cadv}=-\frac{1}{N_{s2t}}\sum_{i=1}^{N_{s2t}}\log D(Z_{s2t,i})-\frac{1}{N_{t}}\sum_{i=1}^{N_{t}}\log(1-D\left(Z_{t,i}\right)) \end{array}$$

where *D* represents the domain discriminator. For the analogous-target domain data, the labels are consistent with those of the source domain for supervised learning, and the supervised loss $L_{sll}$ is defined as follows:

(9) $$\begin{array}{c} L_{sll}=-\frac{1}{N_{s2t}}\sum_{i=1}^{N_{s2t}}{y_{s2t,i}}^{T}\log G_{c}\left(x_{s2t,i}\right) \end{array}$$

### 3.4. High-Confidence Guided Cross-Content Knowledge Distillation

Based on performing style adaptation, we consider the content differences between the analogous-target domain and the target domain, such as foreground edge details. We optimize the training using a knowledge distillation mechanism with self-supervision. As shown in Figure 2, the knowledge distillation structure includes a teacher network Γ, updated via EMA, and a style-adapted student network *S*, both utilizing the same network structure. We update the student model *S* using backpropagation, while the parameters of Γ at time step *t* are the sliding average of the parameters from *S*:

(10) $$\begin{array}{c} \Gamma^{t}=\beta \Gamma^{t-1}+\left(1-\beta \right)S^{t} \end{array}$$

where β is the smoothing coefficient. Subsequently, we use the current teacher model to generate pseudo-labels, which help the student model continue training on the target domain. To address potential domain shifts in the labels, we apply a high-confidence-driven approach to remove unreliable pseudo-labels. Specifically, we set an empirical threshold $\varepsilon_{thresh}$, and only samples with predicted class probabilities above this threshold are considered training samples. Therefore, through this self-supervised knowledge distillation, the model focuses on learning the content patterns in the data, leading to further performance improvement.

The distillation loss, denoted as $L_{self}$, can be described as follows:

(11) $$\begin{array}{c} L_{self}=-\frac{1}{N_{t^{\prime }}}\sum_{i=1}^{N_{t^{\prime }}}{{\tilde{y}}_{t^{\prime },i}}^{T}\log G_{c}\left(x_{t^{\prime },i}\right) \end{array}$$

where $N_{t^{\prime }}$ represents the total number of high-confidence pseudo-labeled samples, and $x_{t^{\prime },i}$ and ${\tilde{y}}_{t^{\prime },i}$ represent the *i*-th target sample and its pseudo-label, respectively.

### 3.5. Overall Training Objective

The overall optimization objective can be divided into two parts: the pre-training part and the style-to-content adaptive part. In the pre-training part, we achieve optimization through the loss function $L_{pre}$. The style-to-content adaptive part consists of cross-style loss and cross-content loss. The cross-style loss includes style supervised learning loss $L_{sll}$, conditional adversarial loss $L_{cadv}$, and prototype constraint learning loss $L_{pll}$, while the cross-content loss is given by $L_{self}$. Therefore, the overall style-to-content adaptive loss can be formulated as follows:

(12) $$\begin{array}{c} L_{FSSC}=\lambda_{1}L_{sll}+\lambda_{2}L_{cadv}+\lambda_{3}L_{pll}+\lambda_{4}L_{self} \end{array}$$

where $\lambda_{1}$, $\lambda_{2}$, $\lambda_{3}$, and $\lambda_{4}$ are the loss balancing coefficients. In particular, to maintain the overall quality of pseudo-labels during training, we only perform cross-style learning before the *q*-th epochs.

## 4. Experiment

### 4.1. Datasets and Metrics

**Figure 5:**
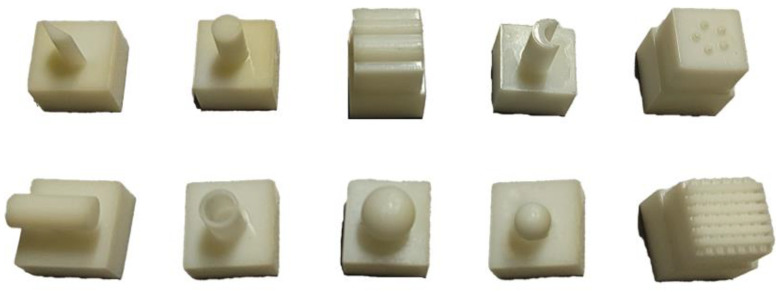
Ten categories of 3D-printed objects for tactile dataset collection. First row: triangle, cylinder, wave, moon, dots; second row: side cylinder, shell cylinder, large sphere, small sphere, crossed lines.

Based on the tactile data collection methods described in [4], we used a standard GelSight tactile sensor, which was fixed to a 3D printer, to touch 10 different 3D-printed objects as a benchmark setup, as shown in Figure 5. Each category contained tactile images of size 224×224 and corresponding labels, captured from a 4×4 grid of planar locations and 11 distinct contact forces. Therefore, a source domain dataset consisting of 1760 images was obtained and named Gel_base. Additionally, we equipped the robotic gripper with three different tactile sensors, a self-made GelSight, a DIGIT, and a 9DTact, to touch the objects, resulting in three target domain datasets, labeled as Gelsight, DIGIT, and 9DTact, respectively. For each target domain dataset, we obtained 48 images per object category from a 4×4 grid of planar locations and 3 distinct contact forces, resulting in a total of 480 images. These images were randomly split into 300 unlabeled samples for the training set and 140 labeled samples for the test set. We also applied several data augmentation techniques, including random cropping, slight random rotations, and random horizontal flips, for training models. Using the four tactile datasets above, we constructed three cross-sensor domain adaptation tasks: Gel_base→Gelsight, Gel_base→DIGIT, and Gel_base→9DTact. We used the common performance metric for classification models, which is classification accuracy. It represents the ratio of correctly predicted samples to the total samples in the test dataset.

### 4.2. Implementation Details

In our proposed method, the feature dimensions of each layer in the SwinT blocks are $\left[96,192,384,768\right]$. The encoder outputs features with a dimension of 768, while the bottleneck layer outputs features with a dimension of 256. The discriminator consists of two [Linear, BatchNorm1d, ReLU] layers followed by a linear layer with a sigmoid activation function.

All algorithms are implemented on the PyTorch platform and trained on a system equipped with a 3.6 GHz Intel Core i7-9700K CPU (8 physical cores), 32 GB of system memory, and an Nvidia GeForce RTX 2080Ti GPU. During the training phase, we use the SGD optimizer, setting the LambdaLR learning rate decay factor to 0.75 and the initial learning rate to 0.01. The weight decay is set to 0.01. We conduct 3 epochs of pre-training, followed by 30 epochs of cross-style adaptive training (q=30). Subsequently, 10 epochs of joint training are performed. The batch size is set to 32 for the entire training process. We also configure the EMA update parameters as α=0.9 and β=0.9. The high-confidence update threshold is set to $\varepsilon_{\mathrm{thresh}}=0.96$. For the Fourier-style transformation, the kernel scale factor is set to lb=0.05. The loss balancing coefficients are set as follows: $\lambda_{1}=1.0$, $\lambda_{2}=1.0$, $\lambda_{3}=1.0$, and $\lambda_{4}=1.0$.

### 4.3. Comparison to State-of-the-Art Methods

We conducted cross-experiment comparisons between FSSC and other state-of-the-art algorithms on three cross-sensor domain adaptation tasks. To ensure fairness, we used the officially released code and default configurations for training.

#### 4.3.1. Quantitative Evaluation

We first evaluated the domain gaps across three pairs of cross-sensor domain adaptation datasets using FID [46], KID [46], and SSIM [47] metrics. In particular, the “Direct” method indicates that the recognition model trained on the standard GelSight dataset was directly evaluated on the self-made GelSight dataset, DIGIT dataset, or 9DTact dataset, respectively. As shown in Table 1, FID and KID indicate that the domain gap between the standard GelSight sensor and the DIGIT sensor was the smallest, while the gap with the 9DTact sensor was the largest, which is consistent with the results of the “Direct” method in Table 2. The larger domain gap observed between the standard GelSight and self-made GelSight datasets, despite originating from the same sensor series, can be attributed to differences in hardware configurations. Variations in elastomer thickness, elasticity, surface properties, and internal illumination conditions during fabrication significantly affected tactile image distributions, resulting in higher FID and KID values. In contrast, the smaller gap between the standard GelSight and DIGIT datasets stemmed from their alignment in tactile image characteristics, such as similar texture distributions, despite differences in base color styles. Therefore, FID and KID provide a quantitative basis for measuring domain gaps between tactile sensor datasets. In addition, although SSIM aligned more closely with human perception, it struggled to effectively measure the intrinsic domain gaps relevant to recognition tasks.

As shown in Table 2, the experimental results on the Gel_base→Gelsight task indicate that the domain gap between sensors of the same series was similar compared to that between sensors of different series. However, the proposed FSSC still achieved the highest performance, demonstrating its effectiveness even in cross-domain recognition tasks involving tactile sensors from the same series but with different manufacturing processes. On the Gel_base→DIGIT task, directly applying the model trained on Gel_base to DIGIT resulted in a poor performance of 21.8%. Feature-based domain adaptation methods, such as ADDA [48] and SR-AQA [49], showed minor improvements. The CTF-CycleGAN [6] algorithm, which employs pixel-level domain adaptation, also enhanced performance but only reached around 55% accuracy. In contrast, our proposed FSSC significantly improved accuracy, outperforming the second-best SR-AQA by approximately 35%. This highlights the limitations of other methods in handling large domain gaps, whereas our method effectively bridged the gap by decoupling style and content, thereby achieving superior results. Similarly, to further validate the cross-domain classification performance of our method on different sensor series, we evaluated FSSC on the Gel_base→9DTact task. FSSC achieved the highest accuracy, significantly outperforming other methods. This further demonstrates the effectiveness of our algorithm in scenarios with substantial domain gaps.

We also conducted fully supervised training in the target domain for three tactile sensors using the backbone network of the proposed method. Specifically, we increased the number of labeled target samples to match the training size on the standard GelSight, defining this as the “Target-Only” method. As shown in Table 2 and Table 3, FSSC demonstrated a slight performance drop in the Gel_base→DIGIT task while using only one-sixth of the unlabeled target samples, effectively reducing annotation costs. In the other two tasks, FSSC achieved the smallest performance drop, outperforming other methods. The “Target-Only” method showed relatively lower performance on 9DTact, which may have arisen from blurred foreground edges in its images caused by sensor fabrication, affecting the recognition of similar objects. In addition, FSSC showed a larger drop in the Gel_base→Gelsight task. We infer that this may have resulted from the larger domain gap, as above discussed, which increased the difficulty of cross-sensor domain alignment and impacted model performance. Overall, although FSSC performed slightly worse than the “Target-Only” method in three tasks, it achieved effective cross-sensor domain adaptation without requiring extensive annotated target domain data.

**Figure 6:**
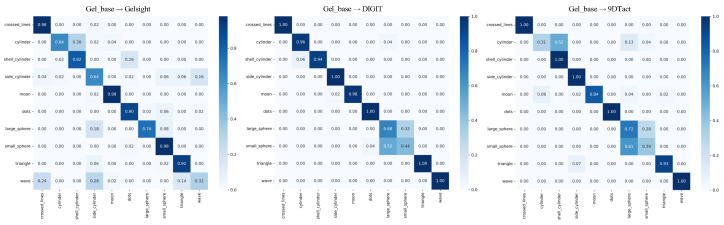
The confusion matrix results of the proposed FSSC method across the three cross-sensor domain adaptation tasks. From left to right, they correspond to the Gel_base→Gelsight, Gel_base→DIGIT, and Gel_base→9DTact tasks, respectively.

Additionally, we computed the confusion matrices for the three tasks, as shown in Figure 6. The confusion matrix provided a detailed correspondence between the true and predicted labels, highlighting the model’s performance across different categories and its error patterns. The confusion matrices indicate that the proposed FSSC achieved high classification accuracy for most categories, particularly excelling in the “Crossed Lines” and “Dots” categories. The diagonal elements for these categories were close to their total counts in the dataset, reflecting the model’s strong predictive capability. In addition, the model performed poorly on the “Large Sphere” and “Small Sphere” categories, primarily because these two objects exhibited highly similar tactile images, making accurate recognition challenging even for humans.

#### 4.3.2. Qualitative Evaluation

**Figure 7:**
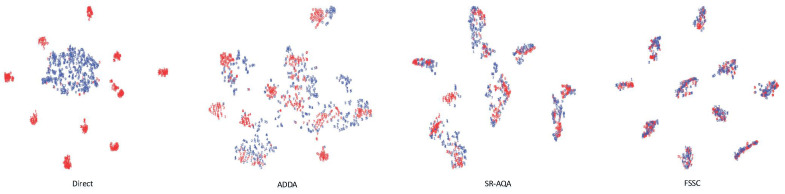
Feature dimensionality reduction visualization results for different algorithms on the Gel_base→DIGIT cross-domain task. The red and blue colors denote the features of the source domain and target domain, respectively. The results are presented from left to right for the “Direct”, ADDA, SR-AQA, and FSSC algorithms.

We utilized t-SNE to visualize the features extracted by the encoders of different algorithms. As shown in Figure 7, the original source and target domain feature distributions exhibited significant differences: source domain features formed multiple scattered clusters, while target domain features remained concentrated in a single cluster. Other comparative methods showed a noticeable alignment between the two domains. However, their class-wise alignment remained suboptimal. In contrast, our proposed FSSC algorithm effectively aligned features across all classes more uniformly, leading to superior performance.

#### 4.3.3. Inference Time

**Figure 8:**
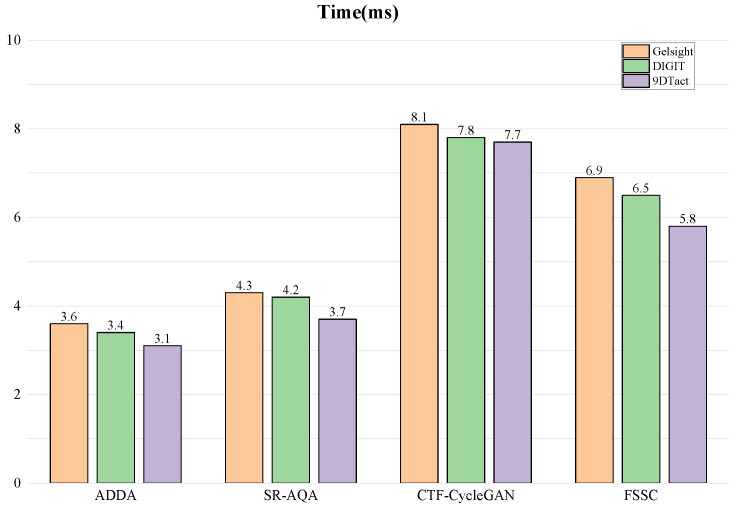
The average processing time for each algorithm during the inference stage.

We compared the inference time of different algorithms across three tasks. During the inference process, we calculated the average processing time for each tactile image. As shown in Figure 8, our algorithm showed no significant increase in time when compared to other feature-level algorithms. Compared with the pixel-level domain adaptation method CTF-CycleGAN, the inference time of our FSSC method was slightly shorter. Furthermore, the average processing time of the FSSC method across the three tasks was 6.4 ms, achieving real-time performance, which makes it suitable for extension to other robotic tasks.

### 4.4. Analysis

We first conducted ablation experiments to validate the contributions of each proposed module, including GLAB, FST, prototype constraint, and the knowledge distillation module. We denote these four ablated versions as w/o GLAB, w/o FST, w/o PC, and w/o KD, respectively. We then explored the impact of the number of target domain training samples on the model and performed a sensitivity analysis of the relevant parameters. Specifically, we focused on the Gel_base→DIGIT task for the ablation study mainly because DIGIT, belonging to a different tactile sensor family, provides a more representative evaluation of our method’s cross-sensor domain adaptation. Additionally, the 9Dtact sensor, with its simple internal light source designed for force estimation, differs from the colorful texture information used in most tactile sensor research. As a result, we selected DIGIT as the target sensor for the ablation study. Additionally, as indicated by the results in Table 1 and Table 2, the final performance of the cross-sensor domain adaptation of our proposed method showed some association with original domain gaps, but high accuracy was achieved in all tasks. Therefore, we infer that conducting ablation experiments with the other two tactile sensors, while potentially showing some differences in recognition accuracy during the ablation process, is still expected to yield similar analytical results.

#### 4.4.1. Module Effectiveness Validation

We conducted ablation experiments on the four proposed modules, and the results are shown in Table 4. After ablating each of the four modules, the classification performance decreased in all cases. The knowledge distillation module had the greatest impact on performance, which also highlights that FSSC decoupled the style and content in domain gaps, enabling the model to more effectively leverage knowledge transfer and handle large domain gaps.

#### 4.4.2. Impact of Target Domain Training Sample Size

To investigate the effect of different target domain training sample sizes on model performance, we selected five different sample size configurations. Specifically, we collected an additional 200 DIGIT tactile images. The experimental results are shown in Table 5, and they indicate that as the sample size increased, the performance continued to improve. However, the performance showed a slight decline between 400 and 500 images compared to 300 images. We infer that this phenomenon may have arisen from changes in data distribution characteristics in unsupervised cross-sensor domain adaptation. As the amount of unlabeled target domain data increased, the model gained more target domain information but may also have encountered noise or distributional bias, especially with significant domain gaps. This could have resulted in a more dispersed distribution, complicating alignment and impacting performance. Furthermore, larger target data volumes may shift the model’s focus to local target features, reducing its generalization to shared features between domains. FSSC can leverage the GLAB layer to extract more compact and essential features, enabling FST-based style transformation and domain-adversarial learning with only a few target domain samples. By eliminating the influence of low-frequency style factors and incorporating high-confidence knowledge distillation, the network can efficiently capture and align the key gaps between domains. Therefore, we ultimately selected 300 target domain images for performance evaluation in our experiments.

#### 4.4.3. Parameter Sensitivity Analysis

**Figure 9:**
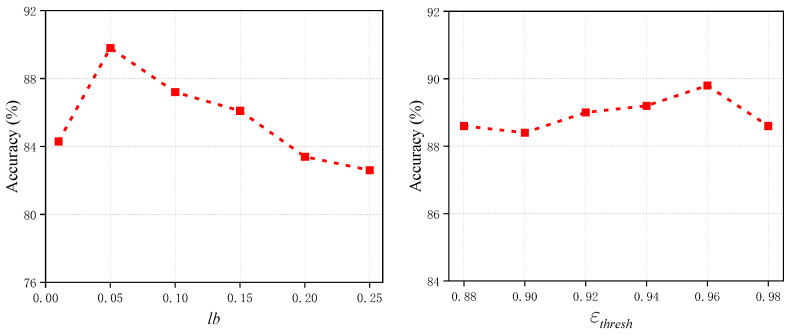
Parameter sensitivity variation curve.

We also conducted a sensitivity analysis of the relevant parameters, including the mask kernel size in the Fourier transform and the high-confidence threshold. As shown in Figure 9, we found that the model achieved the best performance when lb=0.05. We analyze that this scale appropriately transformed the low-frequency information, which aligns with the empirical values for the tactile sensor modality. Furthermore, when the high-confidence threshold was set to $\varepsilon_{thresh}=0.96$, the model achieved the best performance, representing a balanced threshold. Specifically, changes in performance near this threshold were minimal, which indirectly indicates that the choice of this threshold is robust for different sensors.

### 4.5. Robotic Cross-Sensor Tactile Shape Recognition Experiment

**Figure 10:**
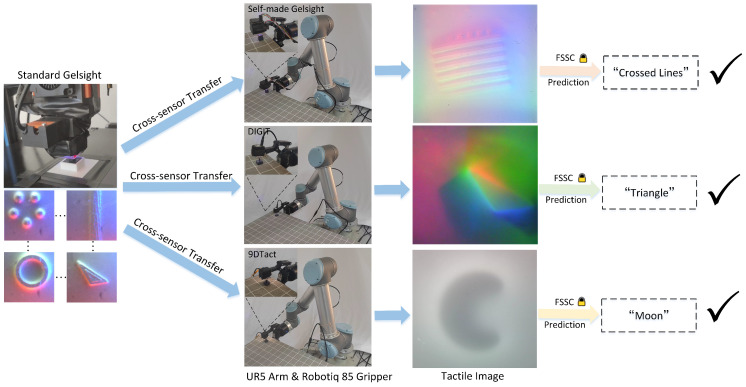
Illustrations of real-world robotic cross-sensor tactile shape recognition results. The frozen FSSC model was trained using sufficient labeled data from the standard GelSight sensor and a few unlabeled target tactile sensor data (self-made GelSight, DIGIT, and 9DTact).

We further equipped the Robotiq 85 two-finger gripper mounted on a real-world UR5 robot with the self-made GelSight, DIGIT, and 9DTact tactile sensors, respectively, to validate the performance of tactile shape recognition in a real-world robotic system, as shown in Figure 10. In the experiments, the robot arm ensured controlled contact force and position, minimizing variations from manual manipulation. In particular, precise control of the applied force was challenging due to the absence of a force sensor. To address this, we approximated the force control using the contact depth between the sensor’s elastomer and the object. Concretely, we used 10 new objects printed with different materials, one from each of the ten categories. We conducted 5 tests for each object, randomly selecting a combination of planar positions from a 4 × 4 grid and 3 vertical positions. The grid center aligned with the sensor’s elastomer center. We used a planar position interval of 2 mm and a vertical position interval of 0.4 mm. The test results are shown in Table 6. The results indicate that the proposed FSSC required only the sufficient standard GelSight dataset and a few unlabeled target tactile sensor data for cross-sensor domain adaptation, achieving up to an 86% recognition success rate on a real-world robotic system equipped with the DIGIT tactile sensor. This effectively validates the cross-sensor tactile shape recognition performance of the FSSC. Notably, the cross-sensor tactile shape recognition results on the three different target tactile sensors are consistent with the cross-sensor domain adaptation outcomes.

### 4.6. Discussion

The key significance of the proposed FSSC lies in its ability to substantially reduce the cost and time required for data collection and annotation in tactile shape recognition tasks. In our experiments, we found that achieving comparable cross-sensor transfer performance of FSSC by training a shape recognition model directly in each target domain would require a dataset size equivalent to that collected using a standard GelSight sensor. In contrast, FSSC achieved similar results with only one-sixth of the samples in each target domain, using exclusively unlabeled data, thus significantly reducing data collection and annotation costs. By leveraging its unsupervised domain adaptation capability, FSSC replaces the high-cost process of collecting large-scale labeled datasets with a more efficient and less labor-intensive collection of a few unlabeled samples. As the complexity of training shape recognition models in target domains increases, the overall cost and time savings offered by this approach become even more pronounced, which highlights its advantages in robotic engineering applications.

## 5. Conclusions and Future Work

This paper proposes a novel few-sample-driven style-to-content unsupervised domain adaptation method, FSSC. The method addresses the significant domain gaps between different sensors by decoupling style gaps and content gaps. FSSC employs FST and prototype-constrained globally conditioned domain adaptation to align style gaps with the target domain. Additionally, high-confidence knowledge distillation for self-supervised learning further aligns content patterns. Moreover, the GLAB layer enhances the network’s feature extraction capabilities. Experiments on three cross-sensor domain adaptation tasks demonstrate the superior performance of our proposed method, achieving the highest accuracy of 89.8% on the DIGIT dataset particularly. An ablation study was also performed to analyze the impact of each component and parameter in our proposed method. Furthermore, when applied to real-world robotic cross-sensor shape recognition, FSSC significantly reduces the reliance on large-scale target domain data, effectively minimizing sensor damage risks and lowering labeling costs. Specifically, with just 300 unlabeled samples from the target domain, it achieved an approximate recognition success rate of 80% across three different sensors.

Currently, the proposed algorithm focuses solely on the fundamental task, i.e., tactile shape recognition. The main considerations are as follows: Recognition tasks serve as a fundamental benchmark for evaluating the effectiveness of domain adaptation techniques in tactile sensing. They enable a rigorous assessment of the proposed method’s ability to address domain gaps without introducing the additional complexity of task-specific variations. Success in recognition tasks can inspire the extension of our approach to more complex tasks. Additionally, this study primarily focuses on domain adaptation within classification tasks, which allows the proposed method to be more easily applied to similar classification tasks than to regression tasks. This constitutes a limitation of the current work. In future research, we intend to broaden the scope of this method to encompass both classification and regression tasks, such as grasp stability evaluation, force estimation, and in-hand object pose estimation, enhancing robots’ manipulation capabilities.

## Figures and Tables

**Table 1:** The results of domain gaps across the three pairs of cross-sensor domain adaptation datasets.

Dataset	FID	KID	SSIM
**Gel_base∪Gelsight**	251.35	18.29	0.67
**Gel_base∪DIGIT**	225.04	16.99	0.58
**Gel_base∪9DTact**	266.73	20.08	0.71

**Table 2:** The accuracy results (%) on the three cross-sensor domain adaptation tasks, with the best-performing method highlighted in bold.

Method	Gel_Base→Gelsight	Gel_Base→DIGIT	Gel_Base→9DTact
**Direct**	17.9	21.8	16.4
**ADDA [48]**	31.2	41.6	33.7
**SR-AQA [49]**	45.0	55.2	49.0
**CTF-CycleGAN [6]**	60.4	54.8	47.6
**FSSC**	**79.2**	**89.8**	**81.2**

**Table 3:** The accuracy results (%) on the three target sensor datasets. The “Target-Only” method indicates that the model was trained and tested solely on the target sensor dataset.

Method	Gelsight→Gelsight	DIGIT→DIGIT	9DTact→9DTact
**Target-Only**	90.6	93.8	87.5

**Table 4:** Ablation experiment results of the proposed different modules, with the best-performing method highlighted in bold.

Method	Datasets	Accuracy (%)
**w/o GLAB**	Gel_base→DIGIT	81.6
**w/o FST**	Gel_base→DIGIT	83.0
**w/o PC**	Gel_base→DIGIT	82.8
**w/o KD**	Gel_base→DIGIT	79.4
**Full**	Gel_base→DIGIT	**89.8**

**Table 5:** Performance results of FSSC with different target domain training sample sizes, with the best-performing method highlighted in bold.

**Sample Size**	100	200	300	400	500
**Accuracy (%)**	74.4	81.2	**89.8**	86.8	87.6

**Table 6:** Robotic cross-sensor tactile shape recognition results.

Test Sensor	#Object	#Attempts	Success Rate (%)
**Self-made Gelsight**	10	38/50	76.0
**DIGIT**	10	43/50	86.0
**9DTact**	10	40/50	80.0

## Data Availability

The data presented in this study are available on request from the authors.
